# CK2 Phosphorylates Sec31 and Regulates ER-To-Golgi Trafficking

**DOI:** 10.1371/journal.pone.0054382

**Published:** 2013-01-18

**Authors:** Mayuko Koreishi, Sidney Yu, Mayumi Oda, Yasuko Honjo, Ayano Satoh

**Affiliations:** 1 The Graduate School of Natural Science and Technology, Okayama University, Okayama, Japan; 2 School of Biomedical Sciences and Epithelial Cell Biology Research Center, The Chinese University of Hong Kong, Hong Kong, People’s Republic of China; 3 The Research Core for Interdisciplinary Sciences (RCIS), Okayama University, Okayama, Japan; Institute of Molecular and Cell Biology, Singapore

## Abstract

Protein export from the endoplasmic reticulum (ER) is an initial and rate-limiting step of molecular trafficking and secretion. This is mediated by coat protein II (COPII)-coated vesicles, whose formation requires small GTPase Sar1 and 6 Sec proteins including Sec23 and Sec31. Sec31 is a component of the outer layer of COPII coat and has been identified as a phosphoprotein. The initiation and promotion of COPII vesicle formation is regulated by Sar1; however, the mechanism regulating the completion of COPII vesicle formation followed by vesicle release is largely unknown. Hypothesizing that the Sec31 phosphorylation may be such a mechanism, we identified phosphorylation sites in the middle linker region of Sec31. Sec31 phosphorylation appeared to decrease its association with ER membranes and Sec23. Non-phosphorylatable mutant of Sec31 stayed longer at ER exit sites and bound more strongly to Sec23. We also found that CK2 is one of the kinases responsible for Sec31 phosphorylation because CK2 knockdown decreased Sec31 phosphorylation, whereas CK2 overexpression increased Sec31 phosphorylation. Furthermore, CK2 knockdown increased affinity of Sec31 for Sec23 and inhibited ER-to-Golgi trafficking. These results suggest that Sec31 phosphorylation by CK2 controls the duration of COPII vesicle formation, which regulates ER-to-Golgi trafficking.

## Introduction

Molecular trafficking and secretion is initiated by the rate-limiting step of protein export from the endoplasmic reticulum (ER). This process is mediated by coat protein II (COPII)-coated vesicles, the formation of which is a crucial step. The COPII coat consists of small GTPase Sar1 and 6 Sec proteins. Coat assembly is initiated by the exchange of GDP for GTP on Sar1 [1], [2], which is catalyzed by the ER-localized guanine nucleotide exchange factor Sec12 [3], [4]. Sar1–GTP recruits the heterodimeric complex Sec23–Sec24 to ER membranes [5], [6], [7]. Sec23 is a GTPase-activating protein (GAP) for Sar1 [8]. Sec24 is thought to bind directly to and sort cargo [9], [10], [11]. The Sec23–Sec24 heterodimers recruited to ER membranes by Sar1–GTP in turn recruit Sec13–Sec31. The Sec13–Sec31 heterodimers form a clathrin-like cage lattice to promote budding of COPII vesicles from ER membranes [12], [13]. Other molecules not included in the COPII coat also modulate COPII vesicle formation [14]. A transmembrane protein, Sec16, has been shown to be necessary for COPII vesicle formation by binding to multiple components of the COPII coat [15], [16], [17], [18], [19], [20].

It is well established that COPII vesicle formation is initiated by the activation of Sar1. However, the regulation of the subsequent process has not been elucidated completely. One way of achieving such a regulation could be Sec31 phosphorylation. Sec31 is phosphorylated in yeast, and its phosphorylation–dephosphorylation cycle is implicated in the budding of COPII vesicles [21]. In addition, mammalian Sec31 has been isolated as a phosphoprotein [22]. However, the molecular nature underlying Sec31 phosphorylation including the responsible kinase(s), phosphorylation sites, and the functional significance in COPII vesicle formation have not been well characterized.

Casein Kinase II (CK2) is a constitutively active serine/threonine kinase that regulates cellular events such as cell cycle and transcriptional regulation, cell survival, virus infection and tumor growth [23]. CK2 is also known as a master kinase that links these cellular events to other kinases [24]. Importantly, the role of CK2 in the regulation of the secretory pathway has been documented by its effect on the trafficking of cystic fibrosis transmembrane conductance regulator (CFTR), mannose 6-phosphate receptor (MPR), and taurine [25], [26], [27]. It is also speculated that CK2 phosphorylates p115, a vesicle tethering factor that is essential for ER-to-Golgi transport [28]. Similar to the proteins described above Sec31 contains multiple CK2 consensus phosphorylation sites. Therefore, we hypothesized that CK2 is responsible for Sec31 phosphorylation, and attempted to determine the role of Sec31 phosphorylation in COPII vesicle formation.

## Results

### Sec31 Phosphorylation Reduces its Membrane Association

**Figure 1:**
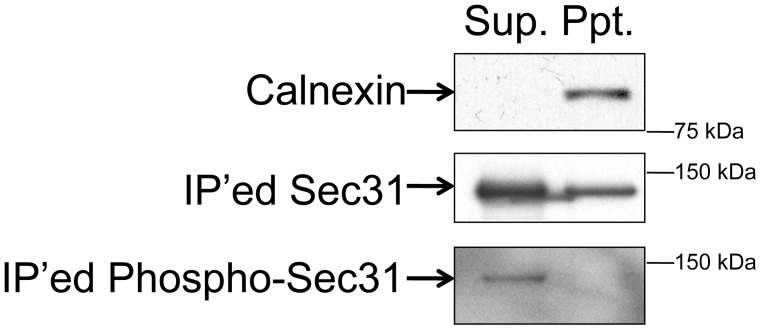
Membrane associated Sec31 is not phosphorylated. HeLa cells were subjected to subcellular fractionations to prepare cytosol (Sup) and membranes (Ppt) by ultracentrifugation. The recovery of the transmembrane protein, calnexin in the membrane fraction (Ppt) indicates that the fractionation was performed properly. Subsequently, Sec31 from both fractions were immunoprecipitated and subjected to western blotting with anti-Sec31 and anti-phospho-serine/threonine antibodies.

To determine the role of Sec31 phosphorylation in COPII-mediated ER-to-Golgi transport, we first examined whether Sec31 phosphorylation affects its membrane association by subcellular fractionation. Ultracentrifugation was used to separate the membranes from the cytosol. Sec31 was recovered from each fraction by immunoprecipitation with anti-Sec31 and its phospho-status was determined by western blotting using anti-phosphoserine/threonine antibodies. As shown in Figure 1, phosphorylated Sec31 was only found in the supernatant (cytoplasmic fraction), whereas Sec31 was detected in both the supernatant and the pellet (membrane fraction; indicated by calnexin). These findings indicate that the phosphorylation of Sec31 reduces its membrane association.

### Sec31 is Phosphorylated at Serines 527, 799, and 1163 and at Threonine 1165

**Figure 2:**
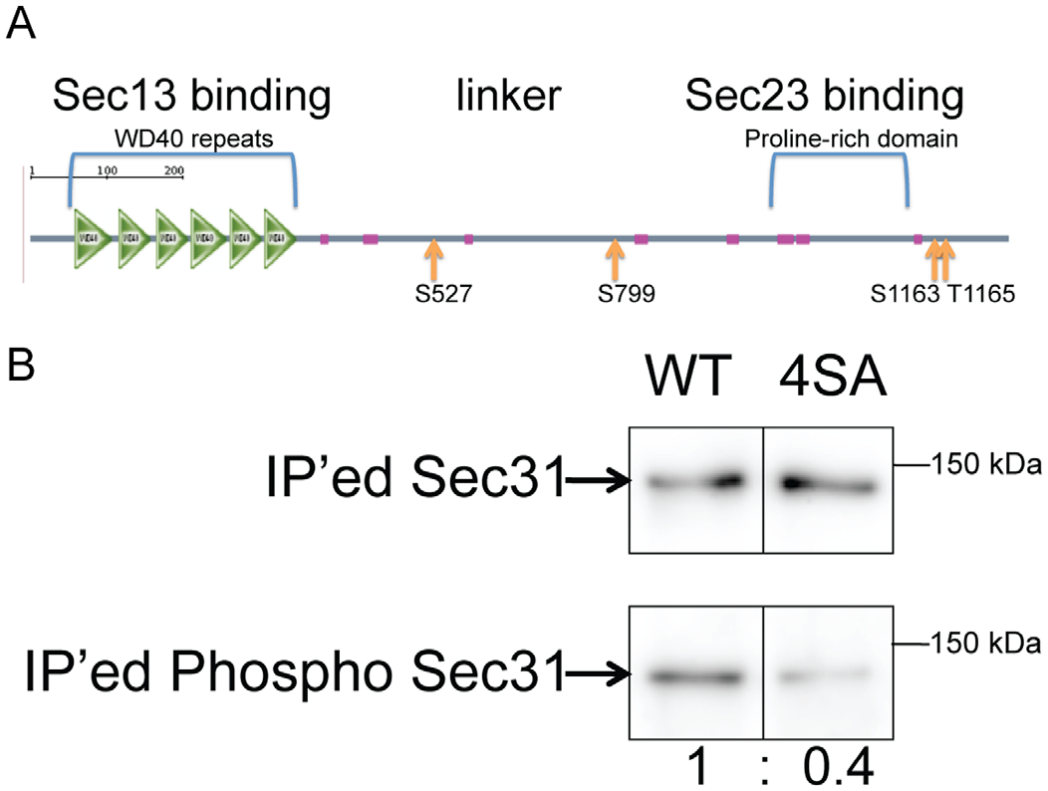
Identification of the phosphorylation sites in Sec31. (A) The predicted domain structure of human Sec31 by SMART (http://smart.embl-heidelberg.de). The domain structure of Sec31 is: N-terminal WD repeats for Sec13 binding, a linker region, and a C-terminal extensin-like proline-rich domain for Sec23 binding. The phosphorylation sites identified by mass-spectrometry were indicated by arrows. (B) HEK293 cells were transfected with FLAG-tagged wild type (WT) Sec31 or its S527A/S799A/S1163A/T1165A mutant (4SA). Tagged proteins were immunoprecipitated with antibodies to the FLAG tag and subjected to western blotting with anti-FLAG and anti-phospho-serine/threonine antibodies. The immunoprecipitated (IP’ed) phospho-Sec31 levels were normalized with IP’ed Sec31 levels and expressed as the normalized ratio.

Since Sec31 phosphorylation appears to be important for its membrane dissociation, we determined its phosphorylation sites. Endogenous Sec31 was isolated from total cell lysates by immunoprecipitation and analyzed by mass spectrometry. Peptide fragments containing serines 527 (S527), 799 (S799), and 1163 (S1163) and threonine 1165 (T1165) were phosphorylated. These sites (indicated by the arrows in Figure 2A) are distributed in the linker region (S527 and S799) and C-terminal Sec23 binding site (S1163 and T1165) of Sec31. To confirm their phosphorylation, the 4 amino acids were mutated to alanines using QuikChange. FLAG-tagged wild-type (WT) and alanine mutant Sec31 (4SA) were expressed and immunoprecipitated from the total cell lysate. Their phosphorylation was analyzed by western blotting using anti-phosphoserine/threonine antibodies. As shown in Figure 2B, the phosphorylation of the 4SA mutant was reduced by approximately 60%. The 4SA mutant was still partially phosphorylated, which suggests that the 4SA mutant is able to form complexes with endogenous WT Sec31 that can be phosphorylated or that there are other phosphorylation sites that were not identified.

### The Non-phosphorylatable Mutant of Sec31 Increases its Membrane Association

**Figure 3:**
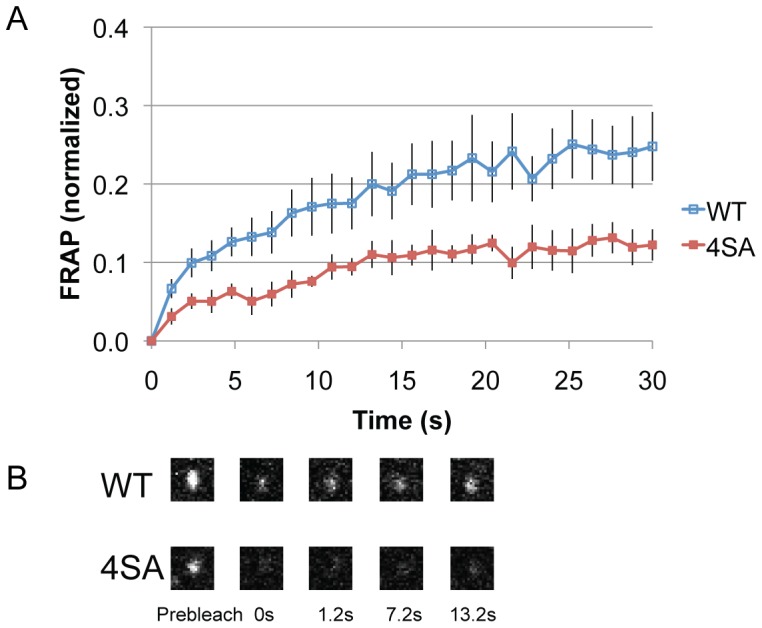
The dynamic of membrane association and dissociation of Sec31 is reduced with the non-phosphorylatable mutant. (A) Shown are the results of fluorescence recovery after photobleaching (FRAP) of Sec31 and its 4SA**.** GFP-tagged wild type Sec31 (WT) and the 4SA mutant were expressed transiently in HeLa cells. A GFP positive dot was photobleached and subsequent fluorescence recovery was monitored for 30 s with 1.2 s intervals. Eight dots were photobleached per cell and results were normalized. Represented are the average of ∼7 experiments. Bars, SEM. (B) The representative images at the indicated time points of Figure 3A. Sizes, 1.85 (w) x 1.85 (h) µm.

Membrane association of the WT Sec31 and the 4SA mutant was assessed by fluorescence recovery after photobleaching (FRAP). GFP-tagged WT Sec31 and the 4SA mutant were expressed transiently in HeLa cells. A GFP-positive dot (an ERES) was then photobleached, and the fluorescence recovery was monitored. As shown in Figure 3, the FRAP of the 4SA mutant was slower than that of the WT. Analysis by fitting the FRAP data to 2 equations (Materials and Methods) showed that the 4SA mutant appeared to have a larger immobile fraction and *k_on_/k_off_* (Table 1). To determine the cause of the difference in the FRAP of WT and 4SA, the FRAP was performed in the presence of cycloheximide. Cycloheximide inhibits protein synthesis resulting in less cargo loading into transport vesicles. Lowering cargo loading by cyclohexmide has been shown to change the turnover of COPII coat [48]. The FRAP of 4SA in the presence or absence of cyclohexmide did not change, suggesting that the difference between WT and SA in Figure 3 may be due to the changes in the turnover rather than lateral diffusion (data not shown). These findings suggest that the 4SA mutant remains on the membranes longer than the WT, which is consistent with the decreased membrane association of phospho-Sec31 shown in Figure 1.

**Table 1:** Kinetics of Sec31 turnover at single ERES.

	Mobilefraction*	t_1/2_ maximumrecovery (s)*	*k_on_/k_off_* **
Wild type (WT)	0.221±0.004	6.13 s ±0.01	8.47±0.14
4SA mutant	0.118±0.002	6.46 s ±0.01	12.32±0.20

* Calculated using the equation 1 in the text and [48].

** Calculated using the equation 2, which is the reaction dominant model described in [49].

### Dephosphorylation of the Linker Region of Sec31 Increases its Binding to Sec23

**Figure 4:**
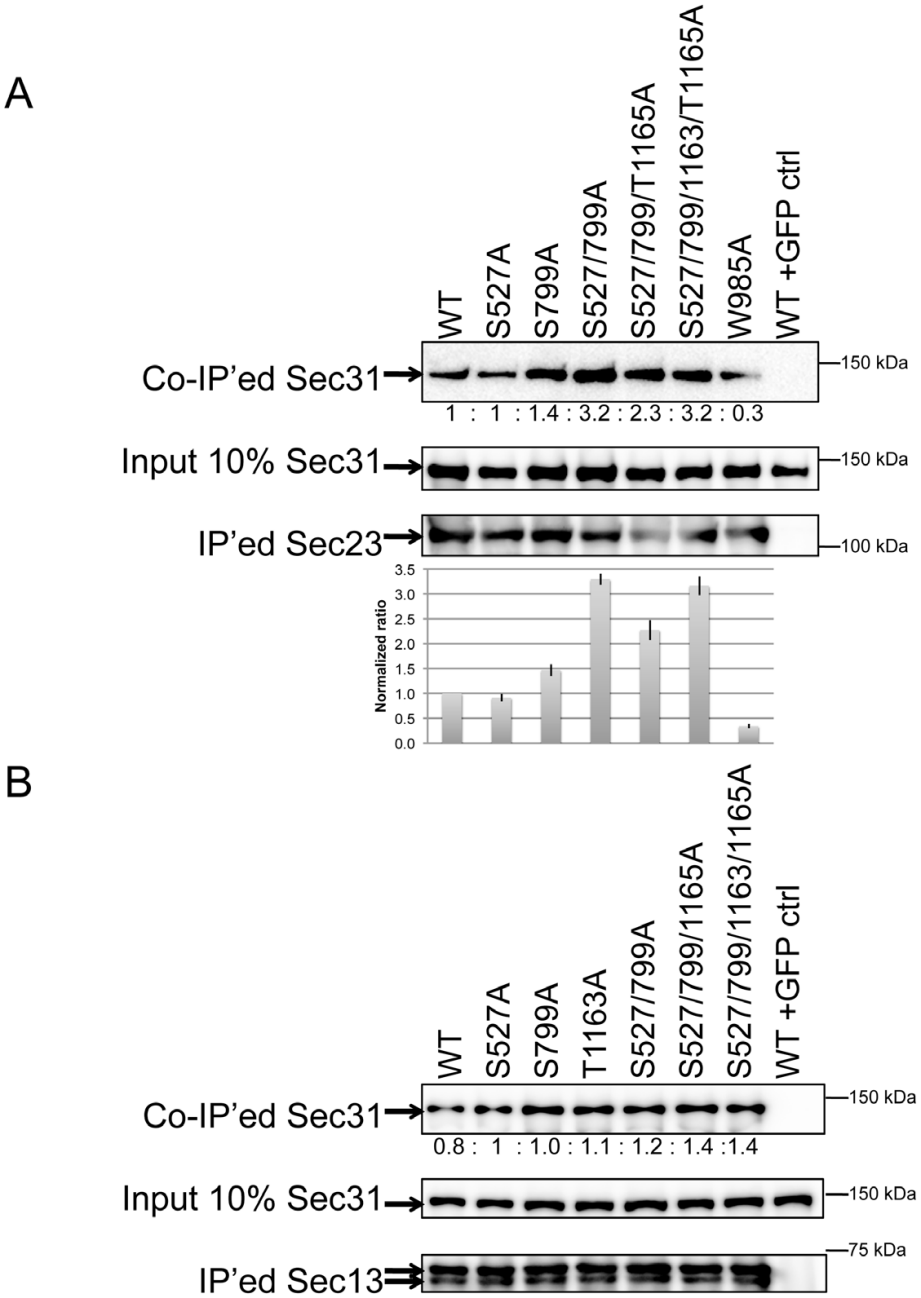
Dephosphorylation of Sec31 at Serines 527 and 799 increases its affinity to Sec23. (A) HEK293 cells were co-transfected with indicated FLAG-tagged Sec31 mutants and GFP-tagged Sec23. Sec23 was immunoprecipitated with anti-GFP beads and subjected to western blotting with anti-FLAG and anti-GFP antibodies. The “input 10% Sec31” is a western blot of 10% aliquots of total cell lysates. The normalized ratio of Sec31 bound to Sec23 was shown below. W985A is a known Sec23-binding defect mutant [52]. The most right lane is a negative control with GFP. The graph below the blots shows the average of the quantification of three independent experiments with SD. (B) FLAG-Sec31 and GFP-Sec13 (instead of GFP-Sec23) were co-expressed and their interactions detected by co-immunoprecipitation and western blotting following the procedures described in Figure 4A. The lane on right most is a negative control with GFP.

Sec23 forms the inner layer of the COPII coat on ER membranes, whereas Sec31 is part of the outer layer. It is thought that Sec31 is recruited to the membrane through direct binding to Sec23 [15], [22]. Therefore, the decreased association of Sec31 with membranes due to phosphorylation led us to examine whether Sec31 phosphorylation also affects its affinity for Sec23. For this purpose, we generated a series of Sec31 non-phosphorylatable alanine mutants. FLAG-tagged Sec31 (WT and alanine mutants) and GFP-tagged Sec23 were then co-expressed, and Sec23 was recovered from total cell lysates by immunoprecipitation with anti-GFP antibodies. As shown in Figure 4A, double mutation of the serines at 527 and 799 (S527/S799) in the linker region led to a 3-fold increase in Sec31 binding to Sec23, whereas there was no or a minimal effect with only a single mutation of S527 or S799, respectively. These effects were specific to Sec23. As shown in Figure 4B, the association of Sec31 with Sec13 was not affected by these mutations. These results indicate that Sec31 phosphorylation regulates its binding to Sec23 but not Sec13. Importantly, the increased affinity of the Sec31 alanine mutants for Sec23 may explain the larger immobile fraction and slower FRAP of the 4SA mutant shown in Figure 3.

### CK2 Phosphorylates Sec31

**Figure 5:**
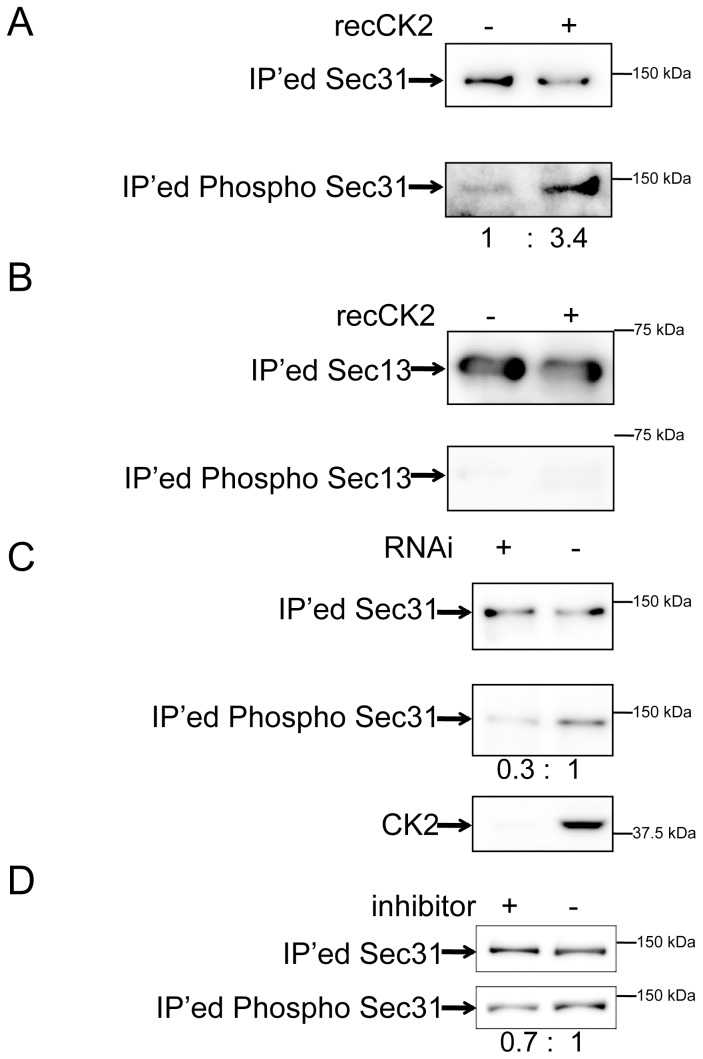
CK2 phosphorylates human Sec31. (A) FLAG-Sec31 expressed in HRK293 cells was immunoprecipitated and incubated with/without recombinant CK2 (recCK2) followed by western blotting with anti-phospho serine/threonine and anti-FLAG antibodies (IP’ed Sec31). The phospho-Sec31 levels were normalized to IP’ed Sec31 levels and expressed as the normalized ratio. (B) FLAG-Sec13 expressed in HRK293 cells was immunoprecipitated, treated and subjected to western blotting as described in (A). (C) Cells were first transfected with siRNA1 to CK2 alpha1 (RNAi +) or to eGFP (RNAi −). After 2 days incubation, cells were transfected with FLAG-tagged Sec31 transfection followed by immunoprecipitation and western blotting as described in (A). Total cell lysates were also analyzed for CK2 by western blotting to determine the depletion efficiency as shown in the bottom panel. (D) FLAG-Sec31 expressed in HRK293 cells treated with or without CK2 inhibitor was immunoprecipitated and subjected to western blotting as described in (A).

Our preliminary data showed that Sec31 was phosphorylated using ^32^P-ATP as well as ^32^P-GTP as a phosphate source (data not shown), which is consistent with the fact that CK2 and CK2-like kinases can use both ATP and GTP as a phosphate source [28]. Therefore, we tested whether CK2 could phosphorylate Sec31. FLAG-tagged Sec31 was expressed, immunoprecipitated from the total cell lysates, and incubated with recombinant CK2 protein (recCK2) before western blotting using anti-phosphoserine/threonine antibodies. recCK2 treatment increased Sec31 phosphorylation more than 3-fold, indicating that CK2 can phosphorylate Sec31 directly at least *in vitro* (Figure 5A). We did not observe phosphorylation of Sec13 treated with recCK2 in the same way suggesting that the phosphorylation of Sec31 by recCK2 is specific (Figure 5B). In addition, recCK2 treatment did not change the weak phosphorylation of 4SA suggesting that there might be additional unidentified phosphorylation sites in Sec31 (data not shown). To confirm whether CK2 is responsible for Sec31 phosphorylation in cells, we depleted CK2 by RNAi. Depletion or inhibition of CK2 reduced Sec31 phosphorylation (Figure 5C and 5D, respectively). These results suggest that CK2 is responsible for Sec31 phosphorylation. The protein levels of CK2 after depletion were 10%–15%, as determined by western blotting using anti-CK2 (Figure S1).

### CK2 Regulates Sec31–Sec23 Interactions Through Sec31phosphorylation

**Figure 6:**
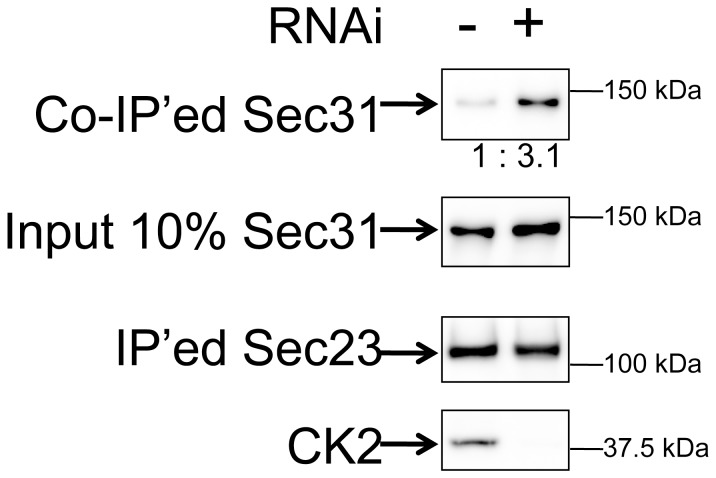
Depletion of CK2 increases Sec31’s affinity to Sec23. Two days before the FLAG-tagged Sec31 and GFP-Sec23 co-transfection, HEK293 cells were transfected with (RNAi +) or without siRNA1 to CK2 alpha1 (RNAi −). GFP-tagged Sec23 was immunoprecipitated with anti-GFP beads and subjected to western blot with anti-FLAG and anti-GFP antibodies. The “input 10% Sec31” is a western blot of 10% aliquots of total cell lysates in order to show the transfection and binding efficiency of Sec31. The co-immunoprecipitated (co-IP’ed) Sec31 were normalized with input Sec31 and expressed as the normalized ratio. In order to check the efficiency of CK2 RNAi, western blotting of the total cell lysates with anti-CK2 antibodies was shown in the bottom panel.

Since Sec31 phosphorylation decreased its binding to membranes and the alanine mutations increased Sec31 binding to Sec23, we predicted that phospho-Sec31 may not bind to Sec23. To test the role of CK2 in Sec31–Sec23 interactions, we manipulated CK2 levels prior to Sec31–Sec23 co-immunoprecipitation in a process similar to that of the experiment shown in Figure 4. As shown in Figure 6, depletion of CK2 by siRNA1 increased Sec31 binding to Sec23 by approximately 3-fold. In contrast, CK2 overexpression decreased such binding (data not shown). We also noticed that CK2 was co-immunoprecipitated with Sec31 in the presence of 1 mM Ca^++^ (Figure S2).

### CK2 Depletion Reduces Membrane Trafficking

**Figure 7:**
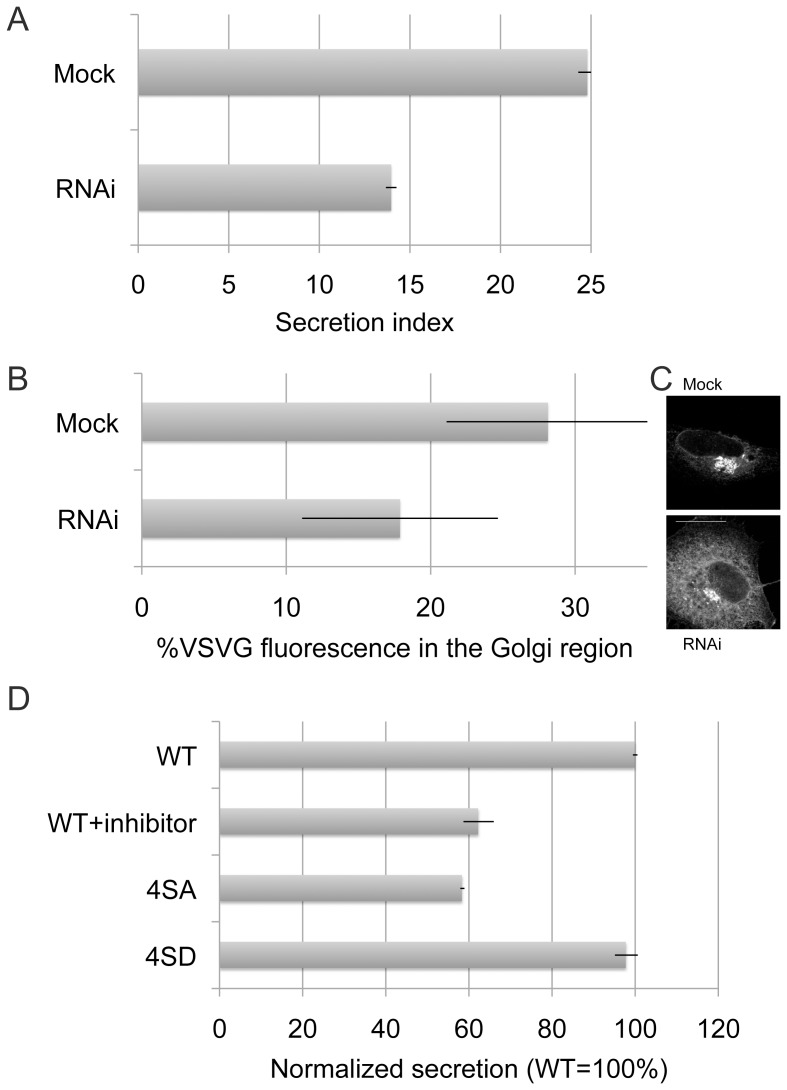
Depletion of CK2 reduces ER to Golgi trafficking. (A) HeLa cells stably expressing secretory alkaline phosphatase (SEAP) were transfected with siRNA1 to CK2 alpha1 (RNAi, 20 nM) or to eGFP (mock). After 90 h, cells were washed and fed with new media. In 6 h, aliquots of culture supernatants were collected and their phosphatase activities were measured in triplicates. Data are presented as a secretion index, which is the ratio of SEAP activity detected in the culture medium to the cellular SEAP activity. Bars, SD (n = 3). (B) CK2 alpha1 siRNA1- or mock-treated cells were transfected with VSV-G-tsO45-YFP plasmid and incubated at the restrictive temperature of 40oC overnight before shifting them to the permissive temperature of 32°C. In 15 min, cells were fixed, permeabilized, and labeled with anti-GM130 antibodies to define the Golgi region. Cell images were captured and analyzed using Image J and Photoshop (n = ∼20 cells). Error bars represent the SEM of three independent experiments. (C) The representative images at the indicated time points of Figure 7B. Bar, 10 µm. (D) HeLa cells stably expressing secretory alkaline phosphatase (SEAP) were transfected with wild type (WT), 4SA mutant (4SA) or 4SD mutant (4SD). After 24 h, cells were washed and fed with new media with or without CK2 inhibitor 1 (Calbiochem). In 6 h, aliquots of culture supernatants were collected and their phosphatase activities were measured in triplicates. Data are presented as a normalized secretion (Secretion index of WT = 100%). Bars, SD (n = 3).

To investigate the function of CK2 in the secretory pathway, we tested whether depletion of CK2 by RNAi affects membrane trafficking. We first monitored the secretion of secretory alkaline phosphatase (SEAP) by measuring SEAP activity in the culture supernatants of mock or CK2 siRNA-treated cells. As shown in Figure 7A, the secretion of SEAP was reduced by approximately 50% in CK2-depleted cells. To confirm the phenotypes of CK2 knockdown by siRNAs, we used siRNA1 and siRNA2. The depletion of CK2 by siRNA1 was more efficient with just 10%–15% of the original CK2 protein level remaining after depletion (Figure S1). The reduction of CK2 by siRNA2 (Figure S3A) was less efficient than that by siRNA1, but the trend in the SEAP assay was the same (Figure S3B). To further determine whether CK2 exerts its effect in ER-to-Golgi transport, we assessed the trafficking of a temperature-sensitive mutant of vesicular stomatitis virus G protein (VSVG). HeLa cells were transfected with VSVG-GFP at 40°C and incubated overnight. After being shifted to 32°C for 15 min, the cells were processed for fluorescence microscopy. The GFP fluorescence intensity in the Golgi region was quantified using Image J software. As shown in Figure 7B, VSVG in the Golgi region was reduced by approximately 40% in CK2-depleted cells. The immunofluorescence images in Figure 7C indicated VSVG is retained in the ER and ERES in CK2-depleted cells. To examine the primary effect of CK2 inhibition in SEAP transport, a CK2 inhibitor was then used. As shown in Figure 7D, the CK2 inhibitor also inhibited SEAP transport similar to CK2 RNAi (Figure 7A). Furthermore, to test if the inhibition of CK2 is primarily caused by inhibition of Sec31 phosphorylation, the SEAP transport was measured in 4SA mutant expressing cells. As shown in Figure 7D, the expression of 4SA mutant reduced SEAP transport by 70%. The 4SD mutant did not affect the transport. These data suggest that CK2 is involved in the regulation of membrane trafficking particularly in ER-to-Golgi transport though Sec31 phosphorylation.

Finally, we measured the colocalization of wild type Sec31 (WT) or 4SA mutant (SA) with Sec24, which is known to colocalize with Sec31 and Sec23 at ERES. As shown in Figure S4, the 4SA mutant colocalized with Sec24 better than the wild type. This is in agreement with our finding that unphosphorylated Sec31 found in the membrane fraction bound better to Sec23 and its FRAP was slower than that of wild type.

## Discussion

COPII vesicle formation is an essential part of protein export from the ER and thus membrane trafficking. However, the regulation of this vital process has not been elucidated completely. In this study, we demonstrated that Sec31 phosphorylation by CK2 plays a crucial role in COPII vesicle formation. Sec31 phosphorylation reduces its association with ER membranes as well as with Sec23. We also identified the Sec31 phosphorylation sites, namely serines 527, 799, and 1163 and threonine 1165, which are located in the middle linker region between the N-terminal WD-40 repeats and C-terminal extensin-like proline-rich domain, and near the C-terminus. The N-terminal WD-40 repeats and C-terminal proline-rich domain of Sec31 have been shown to interact with Sec13 and Sec23, respectively [15], [22]. However, the function of the linker region between the N-terminal and C-terminal domains is unclear. Our results show that a double non-phosphorylatable mutation of S527 and S799 in the linker region markedly increases Sec31 binding to Sec23. This suggests that Sec31 phosphorylation in the linker region changes the conformation of the C-terminal Sec23 binding domain, thereby reducing Sec31 binding to Sec23. In other words, the linker region may be a domain for regulating Sec31 binding to Sec23 through phosphorylation.

We also identified CK2 as a kinase capable of Sec31 phosphorylation. Furthermore, we demonstrated that CK2 facilitates ER-to-Golgi transport and protein secretion. Given that Sec31 membrane association is regulated by its phosphorylation and that CK2 can phosphorylate Sec31, we speculated that the functional role of CK2 in membrane trafficking may be related to its capacity to phosphorylate Sec31 and thus change Sec31 membrane associations. Further studies are required to obtain direct evidence to prove this speculation.

CK2 or a CK2-like kinase has been suggested to be responsible for phosphorylation of the vesicle tethering factor p115 [28]. Similar to Sec31, de-phosphorylated p115 has been shown to be associated with ER membranes [29], although the contribution of p115 phosphorylation to the early secretory pathway may be minimal [30], [31]. p115 phosphorylation has also been implicated in Golgi re-assembly after mitosis. Therefore, our finding represents further evidence of the importance of CK2 for COPII vesicle formation and ER-to-Golgi transport.

Sec31 has been shown to be phosphorylated by several other kinases and several phosphorylation sites have been identified (Table S1) [32], [33], [34], [35]. Four such sites were also identified in our study. We were unable to detect Y804 phosphorylation of Sec31 from HeLa cell lysates by either mass spectrometry or western blotting with anti-phospho-tyrosine antibodies. It is also notable that the region containing S527 and S532 is missing from Sec31 isoform 2, suggesting that CK2 phosphorylation may be only part of the repertoire of kinase regulation of Sec31. In this regard, phosphatases(s) responsible for Sec31 dephosphorylation should be identified to better understand the mechanism of Sec31 phosphorylation. We noticed that CK2 was co-immunoprecipitated with Sec31 in the presence of 1 mM Ca^++^ (Figure S2). This supports our finding that CK2 is a kinase responsible for Sec31 phosphorylation, although the interaction between CK2 and Sec31 may be indirect. Other molecules that have been shown to interact with Sec31, such as ALG-2 and p125, may participate in the CK2–Sec31 interaction and modulate Sec31 phosphorylation by CK2 [36], [37], [38], [39].

Other protein modifications, such as ubiquitination, have also been implicated in the regulation of COPII vesicle formation [40]. Sec31 ubiquitination appears to be important for COPII vesicle formation for large cargo such as collagen. This suggests that ubiquitination may control the size of COPII vesicles. Interestingly, Sec23 binding to Sec31 has been shown to be crucial for collagen trafficking. A mutation in Sec23 at the Sec31 binding site was identified in patients with cranio-lenticulo-sutural dysplasia, which is caused by abnormal collagen trafficking [41], [42], [43].

In conclusion, we found that Sec31 phosphorylation by CK2 decreases its association with ER membranes and Sec23. We also demonstrated that CK2 regulates ER-to-Golgi transport. These findings suggest that CK2 may play an important role in the regulation of COPII vesicle trafficking by the phosphorylation–dephosphorylation cycle of Sec31. Identification of the phosphatase(s) responsible for Sec31 dephosphorylation will help in further understanding the molecular mechanisms underlying the regulation of COPII vesicle formation through Sec31 phosphorylation.

## Materials and Methods

### Antibodies and siRNAs

The antibodies used in this study are as follows: monoclonal anti-casein kinase II, alpha-subunit (anti-CK2; clone 1AD9, Calbiochem, EMD Millipore, Darmstadt, Germany), anti-gamma tubulin (anti-gamma Tub; clone GTU-88) and anti-FLAG tag (anti-FLAG, clone M2, Sigma-Aldrich, St. Louis, MO), anti-GM130 (clone 35), anti-Sec31A (clone 32), anti-phospho-serine/threonine antibodies (anti-pS/pT; clone 22a) and anti-calnexin (clone 37, BD Biosciences, San Jose, CA), anti-Myc tag (anti-Myc, clone 9E10, a gift from Dr. Ira Mellman (Genentech)), polyclonal anti-giantin [44] and anti-GFP [45], Alexa-conjugated secondary antibodies (Invitrogen, Carlsbad, CA), and horseradish peroxidase (HRP)-conjugated secondary antibodies (Pierce, Rockford, IL). Anti-FLAG tag-agarose (M2-agarose) was obtained from Sigma (St. Louis, MO).

The siRNAs used in this study are as follows: siRNA to human casein kinase II alpha 1: siRNA1; CTGGTCGCTTACATCACTTTA (Qiagen, Valencia, CA) and siRNA2; TCAAGATGACTACCAGCTGT (Yale Pathology, New Haven, CT), and siRNA to eGFP: AAGACGTAAACGGCCACAAGTTC (Dharmacon, Thermo Fisher Scientific, Lafayette, CO).

### Cell Culture and Transfection

HeLa or HEK293 cells (CCL-2 or CRL-1573, respectively, ATCC, Manassas, VA) were grown in Dulbecco’s modified Eagle’s medium supplemented with 10% FBS (Invitrogen, Carlsbad, CA). Transient transfection with plasmids and siRNAs were performed using Lipofectamine LTX and RNAiMAX (Invitrogen), respectively, following the manufacturer’s instructions.

### Plasmids and Stable Cell Lines

Plasmids to express secretory alkaline phosphatases (SEAP) and VSV-G-tsO45-SP-YFP (VSVG-YFP), and YFP-Sec23a were kindly provided by Drs. Craig Roy and Derek Toomre (Yale University, CT), and Dr. David Stephens (Bristol University, Bristol, UK). Myc-CK2 alpha 1 was purchased from Origene (Rockville, MD). A cDNA of human Sec31a (clone KIAA 0905) was obtained from Kazusa DNA Research Institute (Kisarazu, Japan). FLAG- or GFP-tagged Sec31 was constructed by subcloning of the human Sec31a cDNA into p3×FLAG-CMV (Sigma) or pQCXIP-GFP (Clontech, Mountain View, CA). Point mutations of Sec31 were introduced using QuikChange kit (Agilent, Santa Clara, CA).

HeLa cells stably expressing SEAP were established as previously described [46].

### Immunoprecipitation, Western Blotting and Cell Fractionation

Cell lysates were prepared with immunoprecipitation buffer (10 mM HEPES-KOH, pH 7.4, 100 mM KCl, 0.1 mM dithiothreitol (DTT), 2.5 mM MgCl_2_, 1% Triton X-100, protease inhibitor cocktails (Roche, South San Francisco, CA) and phosphatases inhibitor cocktails 1 and 2 (Sigma). After incubation for 10 min on ice, the lysate was clarified by centrifugation at 14,000 g for 20 min. For immunoprecipitation, the supernatants were incubated with M2-agarose (Sigma), or anti-Sec31 (Fred Gorelick) or anti-GFP bound to Protein A-Sepharose (GE Healthcare, Piscataway, NJ) for 30 min at 4°C. After washing three times with immunoprecipitation buffer, the samples were fractionated by SDS-PAGE followed by western blotting with antibodies indicated in the figures. Quantification of western blots was performed using Image J software. For some experiments, recombinant CK2 from New England BioLabs (Ipswich, MA) was used to treat the immunoprecipitants for 30 min at 37°C. To identify the phosphorylation sites, immunoprecipitated endogenous Sec31 from total cell lysates was incubated with ^32^P-ATP and cytosol at 37°C for 30 min followed by a separation by SDS-PAGE. Peptides generated by trypsin were analyzed by mass-spectrometry performed by Yale Keck facility (New Haven, CT).

For subcellular fractionation, cells in culture dishes were scraped, suspended in cell fractionation buffer (0.26 M sucrose, 20 mM HEPES-KOH, pH 7.4, 4 mM MgCl_2_, protease inhibitor cocktail and phosphatases inhibitor cocktails 1 and 2) and passed through 25 G needle 6 times using 1 ml syringe, followed by a 4 seconds sonication. The samples were incubated on ice for 20 min, and then centrifuged at 4,400 rpm (2,000 g, A-8-11 rotor, Eppendorf, Hauppauge, NY) for 5 min at 4°C. The supernatants were collected, layered onto 1.2 M sucrose containing Cell fractionation buffer and centrifuged at 55,000 rpm (∼200,000 g) in a TLS-55 rotor (Beckman Coulter, Indianapolis, IN) for 30 min at 4°C. The supernatants and pellets of the 100,000 g spin were collected as the cytoplasmic and membrane fractions, respectively.

### Immunofluorescence Microscopy

Cells on coverslips were fixed with 10% formalin in phosphate buffered saline (PBS) for 15 min, permeabilized with 0.1% Triton X-100 in PBS for 5 min at room temperature. The cells were blocked with 4% bovine serum albumin (BSA) in PBS for 15 min, and then incubated for 15 min with primary antibodies diluted in 4% BSA in PBS. The cells were washed three times with PBS, and incubated for 15 min with secondary antibodies conjugated to Alexa fluorophors (Invitrogen). After washing the cells, the coverslips were mounted on microscope slides and imaged using a FV1000 confocal microscope equipped with a 60× oil objectives (Olympus, Tokyo, Japan). Image data were processed and quantified using Image J software and Adobe Photoshop.

### Fluorescence Recovery After Photobleaching (FRAP)

FRAP assay was performed as described previously [47]. Briefly, HeLa cells on a MatTek glass bottom dish were transfected with GFP-Sec31 wild type or −4SA mutant and incubated overnight. FRAP experiments were performed with a FV1000 confocal microscope (Olympus). After the pre-bleach acquisition, a GFP-Sec31 positive dot was photobleached using the 488 laser at maximum power, and then the fluorescence recovery was followed at 37°C for 30 s with 1.2 s intervals. Eight photobleaches were performed in a cell. Image data were processed and quantified using Image J software. After normalization, the data from 7∼10 experiments were averaged. The analysis of the mobile fraction and of the half time of maximum recovery, and *k_on_/k_off_* of each experiment was carried out after best fitting of the experimental data obtained to a single curve using CurveExpert Pro software (curveexpert.com). The mobile fraction and t_1/2_ maximum recovery (s) were obtained by fitting the FRAP data to the following equation,
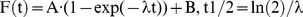
(1)where “A” represents the mobile fraction, “B” is the fluorescence directly after photobleaching (%), and λ is the rate of fluorescence recovery from which t_1/2_ is determined [48]. The *k_on_*/*k_off_* was obtained by fitting the FRAP data to the reaction dominant model,
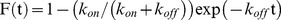
(2)where “*kon*” represents the on rate at the localized binding sites (association rate constant), and “*koff*” represents the off rate at the localized binding sites (dissociation rate constant) [49].

### Secretory Alkaline Phosphatases (SEAP) Transport Assay

SEAP transport assay was performed as previously described [50]. Briefly, HeLa cells stably expressing SEAP were transfected with indicated siRNAs. After 90 h incubation, cells were washed and incubated in fresh culture media for 6 h. The culture supernatant and cells were collected and their SEAP activities were measured using Phospha-light (Roche). Data are presented as a secretion index, which is the ratio of SEAP activity detected in the culture supernatant to that in the cells. In some experiments, CK2 inhibitor (InSolution Casein Kinase II Inhibitor 1, Calbiochem) was used at 1∶1000 dilution.

### Vesicular Stomatitis Virus G Protein (VSVG) Transport Assay

VSVG transport assay was performed as previously described [51]. Briefly, HeLa cells were transfected with siRNA. In 72 h, cells were again transfected with VSVG-YFP plasmid and incubated at the restricted temperature of 40°C overnight. After shifting the permissive temperature of 32°C, cells were incubated for 20 min, and processed for immunofluorescence.

## Supporting Information

Figure S1 **Depletion of CK2 by siRNA1.** HeLa cells were transfected with a siRNA (siRNA1) for CK2 with indicated concentration and the efficiency of CK2 depletion was determined by western blotting. The CK2 protein levels among the samples were normalized by gamma Tubulin protein levels, and quantified by Image J software. The CK2 depletion by CK2 siRNA1 was efficient with 10∼15% remaining CK2 protein level in the experiments shown in Figures 5∼7. Here shows one representative result.
(TIF)

Figure S2 **CK2 interacts with Sec31.** HEK293 cells were co-transfected with Myc-CK2 and GFP-Sec31 or GFP and incubated for 48 h. In the presence of 1 mM Ca^++^ in immunoprecipitation buffer, cells were lysed and GFP-tagged protein was immunoprecipitated with anti-GFP beads and subjected to western blotting with anti-Myc and anti-GFP antibodies. The “input 1% CK2” is a western blot of 1% aliquots of total cell lysates to show the transfection and binding efficiency of CK2.
(TIF)

Figure S3 **Other CK2 siRNA also reduces trafficking.** (A) siRNA2 also reduced CK2 protein level. CK2 protein level was also decreased by siRNA2 but to a less lesser extent (∼50% reduction). (B) Secretory alkaline phosphatase (SEAP) secretion assay using cells transfected with siRNA2. Similar to siRNA1 shown in Figure 7A, reduction of CK2 protein level also reduced SEAP secretion by 75%.
(TIF)

Figure S4 **Sec31 4SA mutant colocalized better with Sec24 than wild type Sec31.** (A) HeLa cells were transfected with GFP-Sec24c and wild type Sec31 (WT) or 4SA mutant (SA). After incubation for 18 h, cells were fixed and stained with immunofluorescence with anti-FLAG shown in red and Hoechst shown in blue. The bottle panels are 4 times magnification of the boxed area of the top panels. Bar, 20 µm. The colocalization of Sec31 and Sec24 were measured using Image J software and shown in (B). Bar, SD.
(TIF)

Table S1 **Phosphorylation sites of Sec31 and experimental conditions in published studies (PMID).**
(DOC)
