# Supplementary figures and images for: CK2 Phosphorylates Sec31 and Regulates ER-To-Golgi Trafficking
Source: PLoS One. 2013 Jan 18;8(1):e54382. doi: 10.1371/journal.pone.0054382 (PMC3548793; doi:10.1371/journal.pone.0054382)

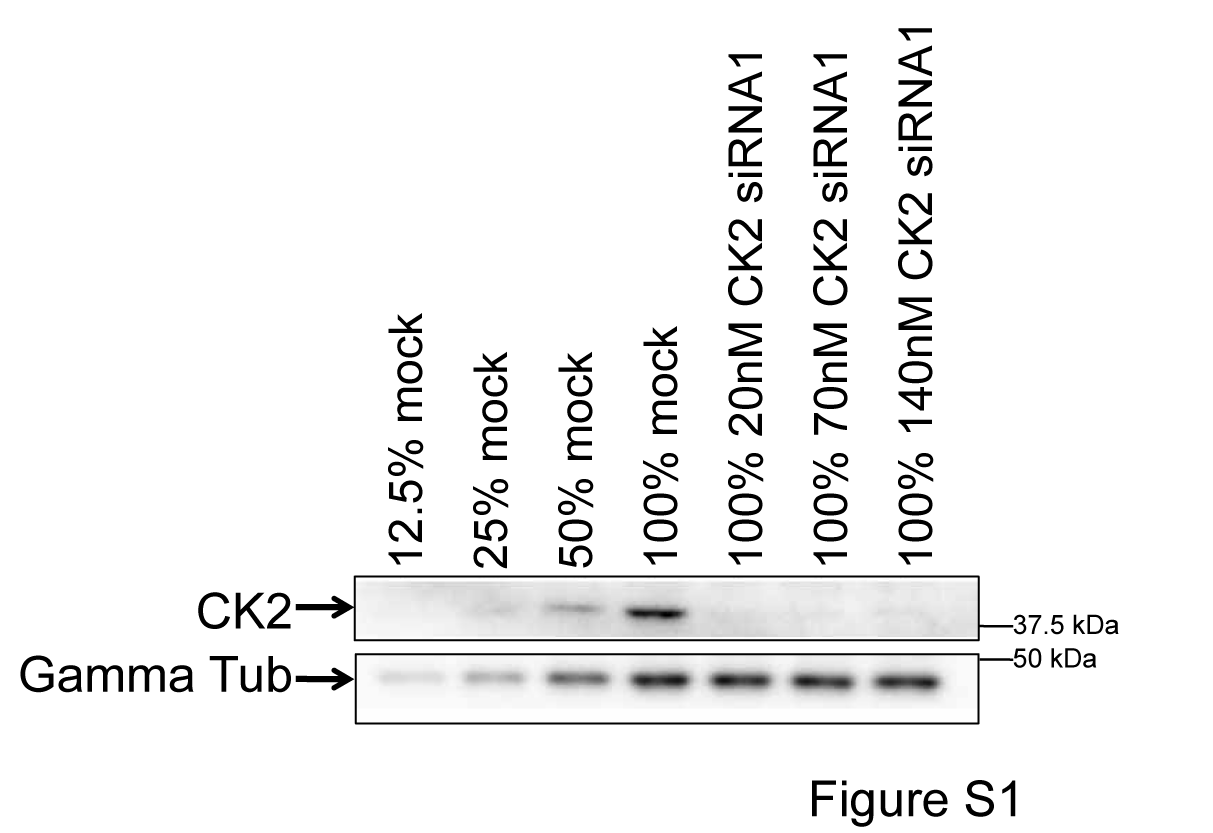

Supplement: Figure S1 — Depletion of CK2 by siRNA1. HeLa cells were transfected with a siRNA (siRNA1) for CK2 with indicated concentration and the efficiency of CK2 depletion was determined by western blotting. The CK2 protein levels among the samples were normalized by gamma Tubulin protein levels, and quantified by Image J software. The CK2 depletion by CK2 siRNA1 was efficient with 10∼15% remaining CK2 protein level in the experiments shown in Figures 5∼7. Here shows one representative result. (TIF) [file pone.0054382.s001.tif]

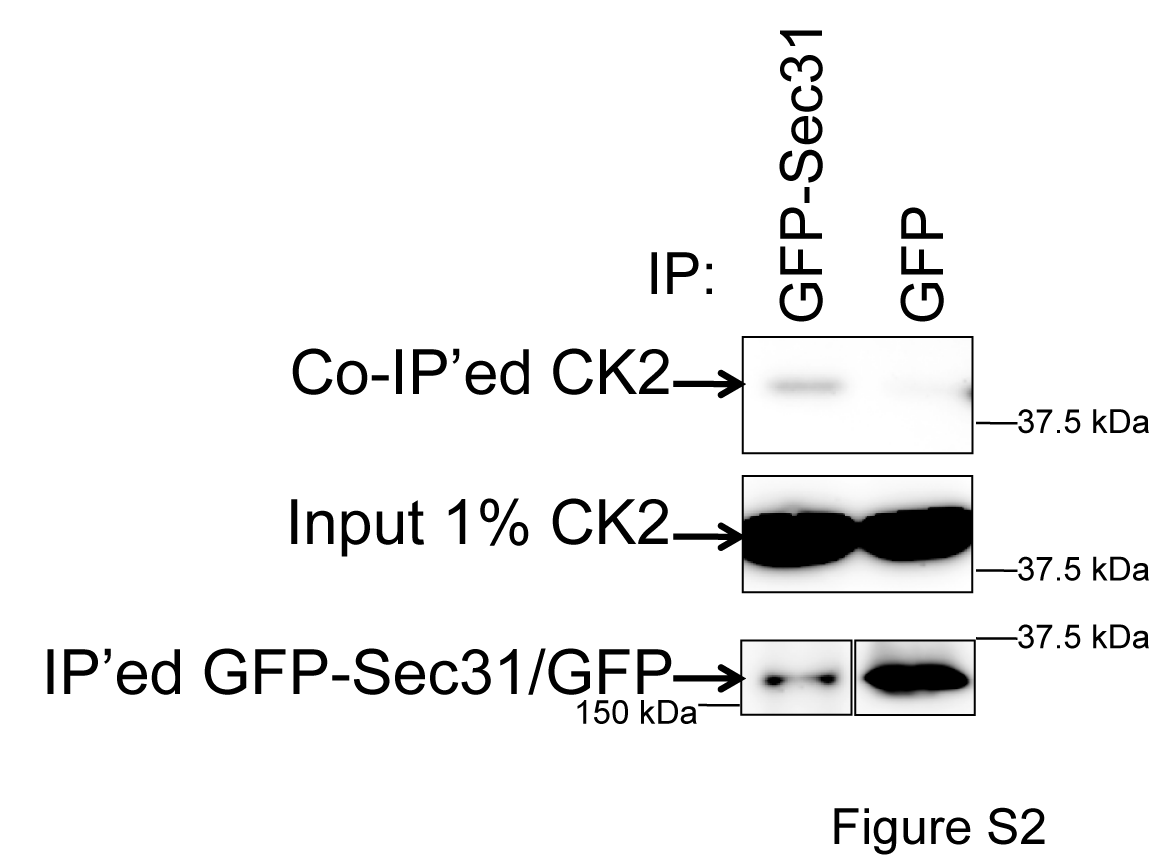

Supplement: Figure S2 — CK2 interacts with Sec31. HEK293 cells were co-transfected with Myc-CK2 and GFP-Sec31 or GFP and incubated for 48 h. In the presence of 1 mM Ca++ in immunoprecipitation buffer, cells were lysed and GFP-tagged protein was immunoprecipitated with anti-GFP beads and subjected to western blotting with anti-Myc and anti-GFP antibodies. The “input 1% CK2” is a western blot of 1% aliquots of total cell lysates to show the transfection and binding efficiency of CK2. (TIF) [file pone.0054382.s002.tif]

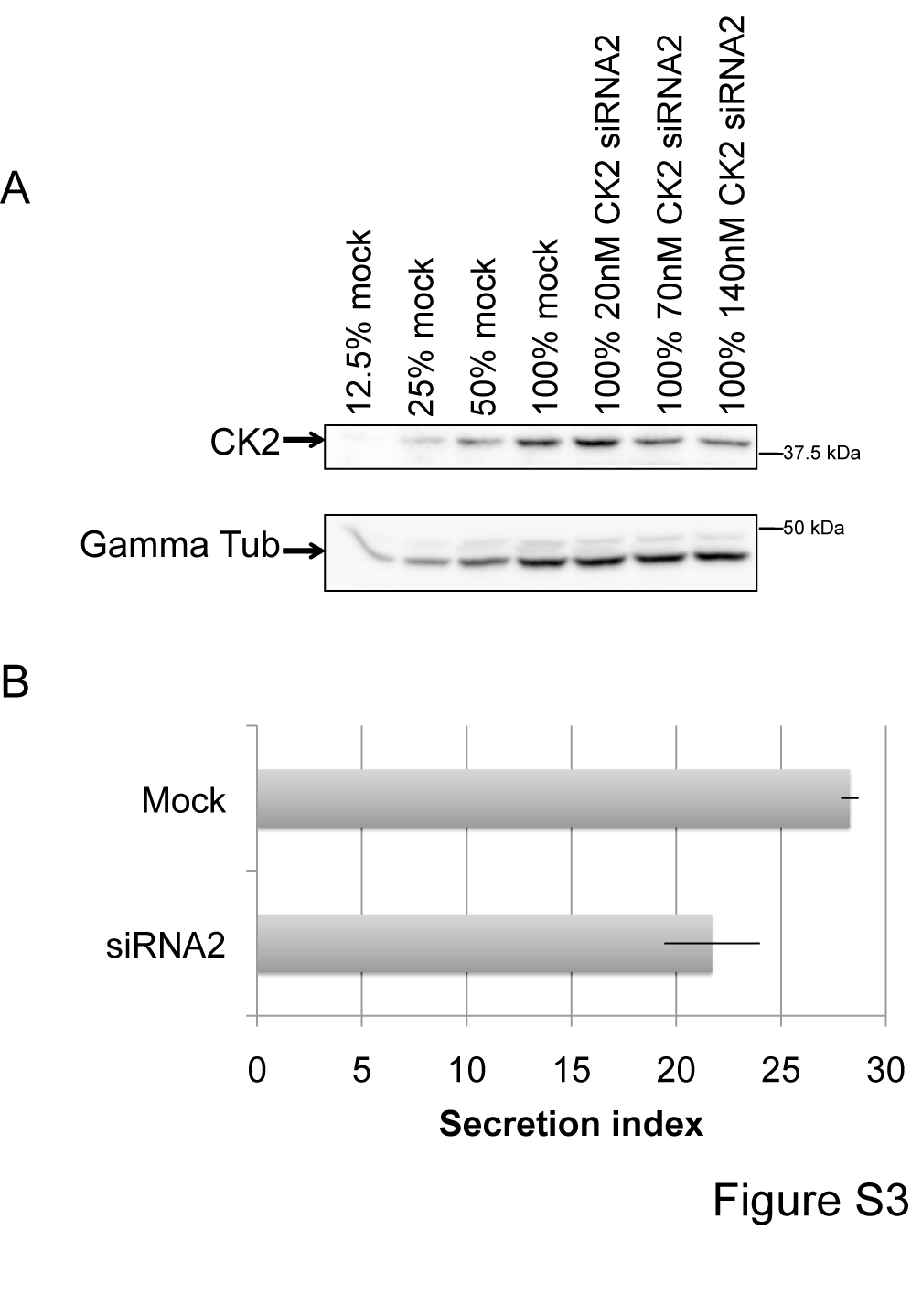

Supplement: Figure S3 — Other CK2 siRNA also reduces trafficking. (A) siRNA2 also reduced CK2 protein level. CK2 protein level was also decreased by siRNA2 but to a less lesser extent (∼50% reduction). (B) Secretory alkaline phosphatase (SEAP) secretion assay using cells transfected with siRNA2. Similar to siRNA1 shown in Figure 7A, reduction of CK2 protein level also reduced SEAP secretion by 75%. (TIF) [file pone.0054382.s003.tif]

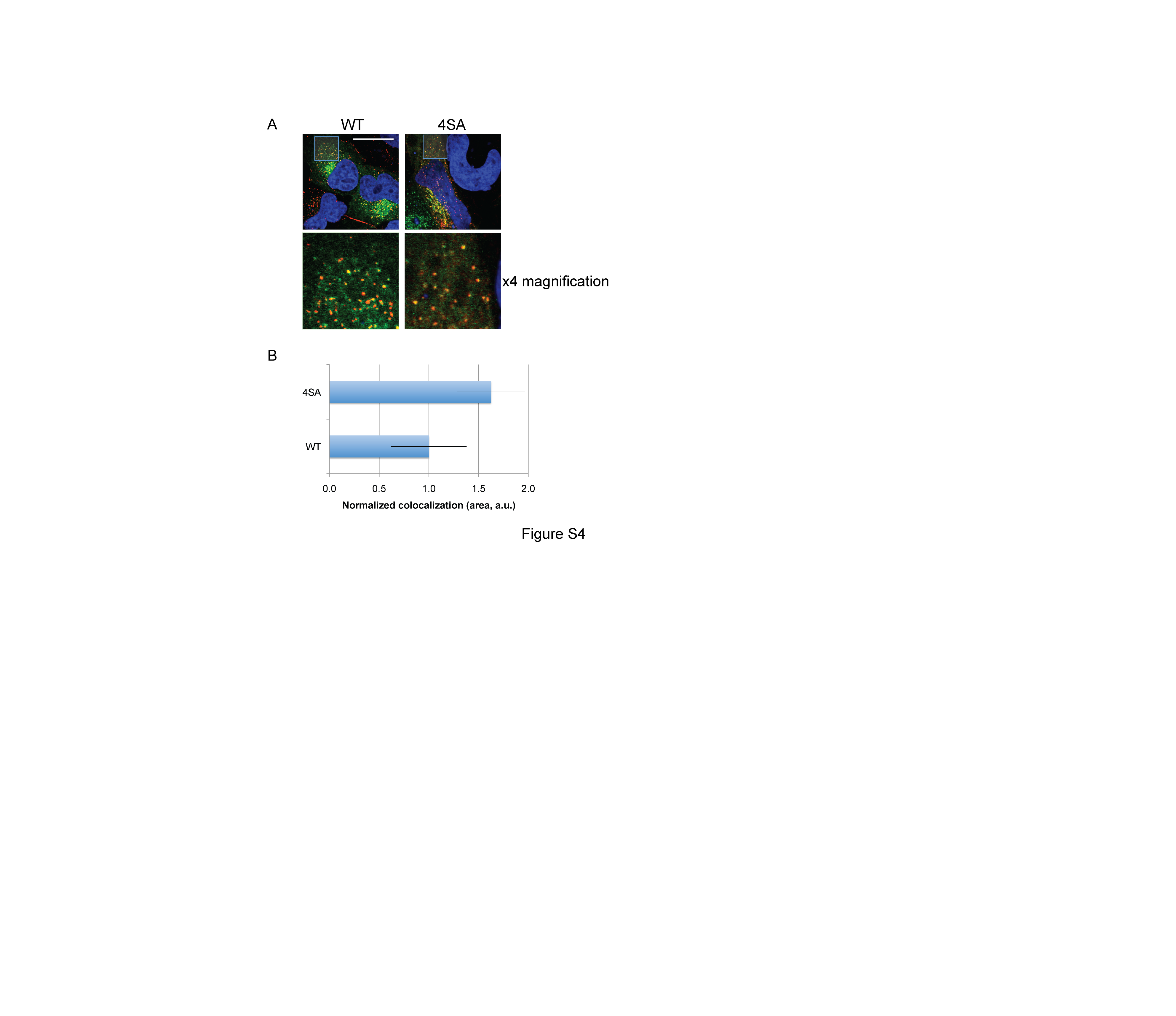

Supplement: Figure S4 — Sec31 4SA mutant colocalized better with Sec24 than wild type Sec31. (A) HeLa cells were transfected with GFP-Sec24c and wild type Sec31 (WT) or 4SA mutant (SA). After incubation for 18 h, cells were fixed and stained with immunofluorescence with anti-FLAG shown in red and Hoechst shown in blue. The bottle panels are 4 times magnification of the boxed area of the top panels. Bar, 20 µm. The colocalization of Sec31 and Sec24 were measured using Image J software and shown in (B). Bar, SD. (TIF) [file pone.0054382.s004.tif]
