# Supplementary material for: CK2 Phosphorylates Sec31 and Regulates ER-To-Golgi Trafficking
Source: PLoS One. 2013 Jan 18;8(1):e54382. doi: 10.1371/journal.pone.0054382 (PMC3548793; doi:10.1371/journal.pone.0054382)
Supplement: Table S1 — Phosphorylation sites of Sec31 and experimental conditions in published studies (PMID). (DOC) [file pone.0054382.s005.doc]

**Table S1 Phosphorylation sites of Sec31 and experimental conditions in published studies (PMID).**

| PMID | Phosphorylation sites | | | | | | | Conditions |
| --- | --- | --- | --- | --- | --- | --- | --- | --- |
|  | S527*^§^ | S532* | S799 | Y804 | T1161 | S1163 | T1165 |  |
| 22496350 [32] | **+** | **+** | **+** | **-** | **-** | **-** | **-** | PKD activation |
| 18669648 [33] | **-** | **-** | **+** | **-** | **-** | **+** | **+** | Mitosis |
| 17081983 [34] | **+** | **+** | **+** | **+** | **+** | **-** | **-** | Stimulation by EGF |
| This study | **+** | **-** | **+** | **-** | **-** | **+** | **+** |  |

*Not available in human Sec31 isoform 2

^§^CK2 consensus site, S/TxxD/E
